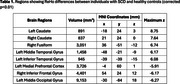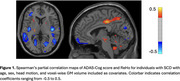# Alterations in resting state regional homogeneity among individuals with subjective cognitive decline and its predictive value

**DOI:** 10.1002/alz70856_102411

**Published:** 2025-12-25

**Authors:** John Nguyen, Tara Riddle, Daniel A. Llano, Brad Sutton, Bharat Biswal, Zhaoyue Shi, Alzheimer's Disease Neuroimaging Initiative

**Affiliations:** ^1^ Clinical Imaging Research, Carle Foundation Hospital, Urbana, IL, USA; ^2^ Department of Neurosciences, Carle Foundation Hospital, Urbana, IL, USA; ^3^ Carle Illinois College of Medicine, University of Illinois Urbana Champaign, Urbana, IL, USA; ^4^ University of Illinois, Urbana Champagne, IL, USA; ^5^ University of Illinois, Urbana, IL, USA; ^6^ Department of Biomedical Engineering, New Jersey Institute of Technology, Newark, NJ, USA; ^7^ University of California San Francisco (UCSF), Los Angeles, CA, USA; ^8^ Laboratory of Neuro Imaging (LONI), University of Southern California, Los Angeles, CA, USA; ^9^ University of San Francisco, San Francisco, CA, USA; ^10^ ADNI, Los Angeles, CA, USA; ^11^ Alzheimer's Disease Neuroimaging Initiative, Washington, WA, USA

## Abstract

**Background:**

Subjective cognitive decline (SCD) is one of the earliest noticeable symptoms of AD and is defined as having self‐perceived worsening of cognition without exhibiting objective impairments on standardized clinical cognitive tests (PMID: 31958406). Since not all individuals with SCD will convert into MCI or AD, it is important to accurately characterize and model the subtle changes in SCD brains that may be predictive of future trajectory towards AD.

**Method:**

Longitudinal MRI data and AD Assessment Scale (ADAS‐Cog) scores from 70 subjects with SCD and 100 healthy controls (HC) were acquired from the ADNI 2 and 3 database. Standard resting‐state fMRI pre‐processing and calculations of 3D regional homogeneity (ReHo) based on 27 neighboring voxels was performed using Data Processing & Analysis for Brain Imaging (DPABI) while site effects were minimized via CovBat harmonization (PMID: 37086875). Within a gray matter mask, two sample t‐tests were performed between the HC and SCD groups' ReHo maps using FSL's randomization procedure with 5000 permutations where age, sex, head motion, and voxel‐wise GM volume were included as covariates. Significance levels were adjusted for multiple comparisons using threshold‐free cluster enhancement correction. An ordinary least squares regression (OLS) model was developed for predicting future ADAS‐Cog scores based on baseline (year 0) clinical scores and ReHo values.

**Result:**

At baseline compared to HC, SCD revealed significant ReHo decreases in the frontal and occipital regions along with significant increases in the temporal regions (Table 1). Correlation maps of SCD ReHo and ADAS‐Cog scores indicated negative correlations in the bilateral insula and temporal regions along with positive correlations in the medial parietal regions (Figure 1). The OLS model developed from these features and baseline clinical scores was able to predict ADAS‐Cog scores at year 4 with a goodness‐of‐fit (r^2^) of 0.72 among a randomized 50:50 training and test set. Independently, the model's r^2^ based on baseline clinical scores alone was 0.64.

**Conclusion:**

ReHo reflects neural synchronization within localized regions, providing insights into brain network coherence. Individuals with SCD exhibit significantly different resting‐state ReHo features compared to HCs. With predictive modeling, these ReHo features can help augment the long‐term projection of early AD trajectories.